# The implementation of a streamlined TAVI patient pathway across five European countries: BENCHMARK registry

**DOI:** 10.1007/s00392-025-02638-z

**Published:** 2025-04-22

**Authors:** Francesco Saia, Sandra Lauck, Eric Durand, Douglas F. Muir, Mark Spence, Mariuca Vasa-Nicotera, David Wood, Cristóbal A. Urbano-Carrillo, Damien Bouchayer, Vlad Anton Iliescu, Christophe Saint Etienne, Florence Leclercq, Vincent Auffret, Lluis Asmarats, Carlo Di Mario, Aurelie Veugeois, Jiri Maly, Andreas Schober, Luis Nombela-Franco, Nikos Werner, Joan Antoni Gómez-Hospital, Julia Mascherbauer, Giuseppe Musumeci, Nicolas Meneveau, Thibaud Meurice, Felix Mahfoud, Federico De Marco, Tim Seidler, Florian Leuschner, Patrick Joly, Jean Philippe Collet, Ferdinand Vogt, Emilio Di Lorenzo, Elmar Kuhn, Vicente Peral Disdier, Gemma McCalmont, Radka Rakova, Wilbert Wesselink, Jana Kurucova, Violetta Hachaturyan, Claudia M. Lüske, Peter Bramlage, Derk Frank

**Affiliations:** 1 Department of Cardiology, Policlinico S. Orsola-Malpighi, University of Bologna, Bologna, Italy; 2 Centre for Cardiovascular Innovation, University of British Columbia, Vancouver, BC Canada; 3 Department of Cardiology, University Hospital of Rouen, Rouen, France; 4 Cardiology Department, James Cook University Hospital, Middlesbrough, UK; 5 Cardiology Department, Mater Private Network, Dublin, Ireland; 6 Cardiology Department, Hospital Sindelfingen-Böblingen, Sindelfingen, Germany; 7 Cardiology Department, Hospital Regional Universitario de Málaga, Malaga, Spain; 8 Department of Cardiology, The Clinique de L’Infirmerie Protestante, Lyon, France; 9 Department of Cardiovascular Surgery, University of Medicine and Pharmacy Carol Davila, Bucharest, Romania; 10 Department of Cardiology, Centre Hospitalier Regional Universitaire de Tours, Hôpital Trousseau, Tours, France; 11 Cardiology Department, Montpellier University Hospital, Montpellier University, Montpellier, France; 12 CHU Rennes Service de Cardiologie, Inserm LTSI U1099, Université de Rennes 1, Rennes, France; 13 Cardiology Department, Hospital de la Santa Creu I Sant Pau, Instituto de Investigación Biomédica Sant Pau, Barcelona, Spain; 14 Structural Interventional Cardiology Division, Department of Clinical & Experimental Medicine, Careggi University Hospital, Florence, Italy; 15 Department of Cardiology, Institut Mutualiste Montsouris, Paris, France; 16 Cardiac Center, IKEM Prague, Prague, Czech Republic; 17 Department of Cardiology, Hospital Floridsdorf and the Karl Landsteiner Institute for Cardiovascular and Critical Care Research Vienna, 1210 Vienna, Austria; 18 Instituto Cardiovascular, Hospital Clínico San Carlos, Instituto de Investigación Sanitaria del Hospital Clínico San Carlos (IdISSC), Madrid, Spain; 19 Medical Department III, Heart Center Trier, Krankenhaus der Barmherzigen Brüder, Trier, Germany; 20 Heart Diseases Institute, Bellvitge University Hospital - IDIBELL, L’Hospitalet de Llobregat, University of Barcelona, Barcelona, Spain; 21 Department of Internal Medicine 3/Cardiology, University Hospital St. Pölten, St. Pölten, Austria; 22 Cardiology Division, AO Ordine Mauriziano, Turin, Italy; 23 Cardiology, Besancon Regional University Hospital Center, Besancon, France; 24 Cardiology, Polyclinique Du Bois, Lille, France; 25 Internal Medicine III, Cardiology, Angiology and Internal Intensive Care Medicine, University Hospital of Saarland, Homburg, Germany; 26 Centro Cardiologico Monzino IRCCS, Milan, Italy; 27 Department of Cardiology and Pulmonology, Georg-August-University, Göttingen, Germany; 28 Department of Cardiology, Campus Kerckhoff of the Justus-Liebig-Universität Gießen, Kerckhoff-Clinic, German Centre for Cardiovascular Research (DZHK), Bad Nauheim, Germany; 29 Department of Medicine III, German Centre for Cardiovascular Research (DZHK), University of Heidelberg, Heidelberg, Germany; 30 Department of Interventional Cardiology, Hôpital Saint Joseph, Marseille, France; 31 Department de Cardiologie, Hôpital de Pitié-Salpêtrière AP-HP, Paris, France; 32 Department for Cardiovascular Surgery, Artemed Klinikum München, Munich, Germany; 33 Department of Cardiac Surgery, Paracelsus Medical University, Nuremberg, Germany; 34 U.O.C. Cardiologia/UTIC “d. Rotiroti”, Department of Cardiology and Cardiovascular Surgery, A.O.R.N. San Giuseppe Moscati Avellino, Avellino, Italy; 35 Faculty of Medicine, Department of Cardiothoracic Surgery, Heart Center, University Hospital of Cologne, Cologne, Germany; 36 Cardiology Department, Hospital Universitari Son Espases, Palma, Spain; 37 Grupo de Investigación de Fisiopatología y Terapéutica Cardiovascular, Institut d’Investigació Sanitària Illes Balears (IdISBa), Palma, Spain; 38 Edwards Lifesciences, Prague, Czech Republic; 39 Institute for Pharmacology and Preventive Medicine, Cloppenburg, Germany; 40 Department of Internal Medicine III (Cardiology and Critical Care Medicine), University Clinical Centre Schleswig-Holstein (UKSH), Kiel, Germany; 41 German Centre for Cardiovascular Research, Partner site Hamburg/Kiel/Lübeck, Arnold-Heller Strasse 3, 24105 Kiel, Germany

**Keywords:** Aortic stenosis, Quality of care, Prospective registry, Transcatheter aortic valve implantation, TAVI, Clinical care

## Abstract

**Background:**

Benchmark best practices have been shown to streamline the clinical pathway for patients undergoing transcatheter aortic valve implantation (TAVI), but the impact in diverse health systems is unknown.

**Aims:**

We evaluated the impact of Benchmark best practices implementation in Germany, Austria, France, Spain, and Italy.

**Methods:**

International, multicentre registry of severe symptomatic aortic stenosis (AS) patients undergoing TAVI with a balloon-expandable valve, before and after Benchmark best practices implementation. Objectives were to reduce overall and intensive care unit (ICU) length of stay (LoS), and to document 30-day safety.

**Results:**

A total of 890 patients were analysed in France, 454 in Spain, 362 in Germany, 300 in Italy, and 176 in Austria. Patients had the highest surgical risk in Germany (EuroSCORE II 6.8 ± 7.3%) and lowest in Spain (3.8 ± 2.6%). Austrian patients reported higher rates of prior myocardial infarction, severe pulmonary hypertension, and aortic valve-related symptoms at baseline. After the implementation of Benchmark best practices, the median hospital LoS was significantly reduced in France (5 vs. 3 days, *p* < 0.001), Spain (6 vs. 4, *p* < 0.001), Germany (9 vs. 6, *p* < 0.001), and Italy (7 vs. 5, *p* < 0.001); reductions in median ICU LoS were reported in France (1.1 vs. 0 days, *p* < 0.001), Spain (1.9 vs. 1, *p* < 0.001), and Germany (1 vs. 0.9, *p* = 0.004). Across all countries, 30-day safety outcomes were uncompromised and reduced rates of major vascular complications rates were observed in Germany (5.9 vs. 0.0%, *p* < 0.001).

**Conclusion:**

The implementation of Benchmark best practices in diverse European healthcare systems resulted in reduced hospital and ICU LoS without compromising patient safety.

**Trial registration:**

ClinicalTrials.gov NCT04579445, September 28th, 2020

**Supplementary Information:**

The online version contains supplementary material available at 10.1007/s00392-025-02638-z.

## Introduction

Since the first-in-human transcatheter aortic valve implantation (TAVI) in 2002 in Europe, the procedure has been rapidly adopted outnumbering surgical aortic valve replacement (SAVR), with a predicted further sharp increase due to expanding indications and an ageing population [1, 2]. Currently, an estimated 115,000 candidates are eligible for TAVI per year in Europe alone, with the highest rates predicted in Germany (20,466 candidates), followed by Italy (15,784 candidates), France (14,632 candidates), and Spain (10,274 candidates) [3]. The European Society of Cardiology (ESC) and the European Association for Cardio‐Thoracic Surgery (EACTS) guidelines for the management of valvular heart disease provide detailed guidance on decision-making, including accurate diagnosis, risk assessment, as well as timing and indication for the most suitable type of intervention(s) [4]. However, there are currently no consensus recommendations on how to streamline the patient pathway to optimize timely access to care and mitigate the risks of in-hospital complications [5]. Given the steep increase in aortic stenosis (AS) prevalence and existing variation in regional strategies related to healthcare resources, pre-procedural planning, and patient management, there is a need for a comprehensive approach to standardize quality of care delivered to patients undergoing TAVI.

The European FAST-TAVI (Feasibility And Safety of Early Discharge After Transfemoral [TF] Transcatheter Aortic Valve Implantation) project has previously shown that the use of pre-specified risk criteria effectively guided the timing of discharge and is associated with 1-year safety [6, 7]. In addition, the implementation of a clinical valve coordinator or TAVI nurse led to improved discharge management and increased patient satisfaction without compromising safety [8]. This initiative built on the minimalist Vancouver 3M (multidisciplinary, multimodality but minimalist) Clinical Pathway approach was introduced to standardize care and reduce hospital length of stay (LoS) [9]. The process includes risk-stratified pre- and peri-procedural practices, post-procedure care with early mobilization and reconditioning, and a criteria-driven discharge algorithm.

In this context, the objective of the BENCHMARK registry was to measure the impact of eight best practices on LoS (admission to discharge), duration of critical care, and patient satisfaction with uncompromised patient safety in diverse European countries. At 30 days, the standardized implementation of Benchmark was safe and reduced total hospital and critical care LoS [10].

## Methods/design

BENCHMARK (ClinicalTrials Identifier: NCT04579445) is a multicentre, international study of patients with severe symptomatic AS undergoing TAVI at 28 centres across Europe including Austria, France, Germany, Italy, Czech Republic, Romania, and Spain [11]. For the current analysis, the Czech Republic and Romania were excluded due to low enrolment in single centres. Benchmark was conducted according to the European Medical Device Regulations, the International Organization for Standardization (ISO 14155:2020), and the Declaration of Helsinki. The registry was approved by the independent ethics review boards of participating sites and patients provided written informed consent.

### Patient selection

Between January 2020 and March 2023, the BENCHMARK registry documented a total of 2388 patients. Patients were enrolled retrospectively prior to, and prospectively after the introduction of the Benchmark best practices. Key inclusion criteria were transfemoral TAVI with a balloon-expandable valve, 18 years and older, and scheduled for 30-day and 1-year follow-up visits. Patients were excluded if they were undergoing valve-in-valve procedures, were pregnant, or for whom key variables for the assessment were missing in the retrospective cohort.

### Benchmark best practices

The development of eight best practices was informed by contemporary evidence and expert clinical opinions[12] and included (1) education of patient and family; (2) determination of anticipated discharge date at admission based on pre‐procedural risk stratification and scheduling of post‐procedural diagnostics; (3) echocardiographic‐ or angiographic check at the end of the procedure immediately performed to confirm vascular access closure and absence of peri-procedural complications; (4) nurse-led early mobilization protocol; (5) use of decision tree to determine the need for new pacemaker if required without increasing hospital stay; (6) criteria-based safe discharge home; (7) daily visit by implanting physician and interaction with the rest of the team; and (8) education and alignment of the internal team (medical, nursing, and paramedical). The detailed implementation process, including the education of centres and local planning, has been previously described elsewhere [11].

### Study end points

The reduction in the total hospital LoS (from admission to discharge) and the duration of critical care admission (defined as ICU, cardiac/coronary care unit [CCU] or intermediate care [IMC]) were the primary end points. Secondary objectives included the assessment of procedural success and complications and patient safety at 30 days. While the overall Benchmark programme was powered to ascertain the safety of patients, the comparison of outcomes prior to and following Benchmark implementation across five countries is exploratory.

### Statistical analysis

All available patient data were analysed and missing data were not imputed. Continuous variables were presented as mean ± standard deviation (SD) or median and interquartile ranges (IQR). The Kolmogorov–Smirnov test was used to test for normal distribution. Categorical variables were reported as frequencies and percentages. All analyses were performed using IBM SPSS Statistics version 29 (IBM, Armonk, New York) or R Core Team (https://www.R-project.org/).

## Results

A total of 890 patients in France, 454 in Spain, 362 in Germany, 300 in Italy, and 176 in Austria were included. The implementation rates of Benchmark best practices by country are provided in Supplementary Fig. 1. Baseline patient characteristics by country prior and after the implementation of Benchmark are provided in Supplementary Table 1. The detailed results for each country are presented below.

### France

Patients in France reported the lowest rates of aortic valve-related symptoms at baseline, including dizziness (6.4%), (pre-) syncope (6.0%), and Canadian Cardiovascular Society (CCS) angina class 3/4 (2.1%) (Table 1). Patients treated in France tended to have less comorbid conditions, including prior myocardial infarction (10.5%) and severe pulmonary hypertension (3.5%). Nevertheless, the aorto-ventricular mean pressure gradient (PG) was highest in the French cohort (47.2 ± 12.8 mmHg) (Table 2).

**Table 1:** Patient characteristics in the total patient population by country

Characteristic, *n* (%) or mean ± SD or median (IQR)	France (890 pts, 9 sites)	Spain (454 pts, 5 sites)	Germany (362 pts, 6 sites)	Italy (300 pts, 4 sites)	Austria (176 pts, 2 sites)	*p* value
Age (years)	80.1 ± 7.0 81.0 (76.0, 85.0)	80.7 ± 6.4 81.0 (77.0, 85.0)	79.6 ± 6.6 81.0 (76.0, 84.0)	81.6 ± 4.9 82.0 (79.0, 85.0)	80.1 ± 6.4 81.0 (77.8, 84.0)	< 0.001
Female gender	344 (38.9)	189 (41.6)	133 (36.7)	132 (45.8)	61 (34.7)	0.070
Body mass index (kg/m^2^)	27.6 ± 5.1 27.0 (24.2, 30.5)	28.0 ± 5.3 27.4 (24.7, 30.4)	27.1 ± 4.5 26.6 (24.1, 29.7)	26.8 ± 4.5 26.5 (23.5, 29.1)	27.9 ± 4.8 27.0 (24.7, 30.5)	0.008
Aortic valve-related symptoms						
Dizziness with exertion	56 (6.4)	228 (50.2)	62 (17.1)	60 (21.5)	112 (63.6)	< 0.001
(Pre-)syncope	52 (6.0)	53 (11.7)	40 (11.0)	31 (11.1)	28 (15.9)	< 0.001
NYHA class III or IV	443 (50.8)	262 (57.7)	233 (64.9)	171 (61.3)	107 (60.8)	< 0.001
Angina CCS 3 or 4	19 (2.2)	17 (3.7)	15 (4.2)	24 (8.6)	21 (11.9)	< 0.001
Risk scores and frailty						
EuroSCORE II	4.6 ± 6.5 2.4 (1.6, 5.0)	3.8 ± 2.6 3.4 (2.0, 4.3)	6.8 ± 7.3 4.3 (2.1, 8.1)	6.0 ± 6.5 3.5 (2.2, 6.8)	4.0 ± 3.8 2.8 (1.8, 4.7)	< 0.001
Frailty, severe	11 (1.3)	29 (6.4)	19 (5.2)	5 (1.8)	4 (2.3)	< 0.001
Impaired mobility	52 (5.9)	76 (16.7)	100 (27.6)	14 (5.0)	30 (17.0)	< 0.001
Cognitive deficit	20 (2.3)	23 (5.1)	17 (4.7)	10 (3.6)	6 (3.4)	0.070
Comorbidities						
Prior MI	92 (10.5)	79 (17.4)	42 (11.6)	37 (13.2)	51 (29.0)	< 0.001
Peripheral artery disease	165 (18.8)	43 (9.5)	31 (8.6)	57 (20.3)	31 (17.6)	< 0.001
Diabetes mellitus	229 (26.1)	194 (42.7)	111 (30.7)	75 (26.7)	65 (36.9)	< 0.001
PH, severe^a^	26 (3.5)	30 (7.2)	31 (8.8)	17 (7.2)	21 (15.4)	< 0.001
Renal insufficiency	225 (25.5)	107 (23.6)	95 (26.2)	87 (31.0)	51 (29.0)	0.213
Prior pacemaker	53 (6.3)	31 (6.8)	27 (7.7)	12 (4.4)	14 (8.1)	0.445

*CCS* Canadian Cardiovascular Society, *IQR* interquartile range, *MI* myocardial infarction, *NYHA* New York Heart Association, *SD* standard deviation, *pts* patients, *PH* pulmonary hypertension

^a^Mean pulmonary arterial pressure > 55 mmHg

**Table 2:** Electrocardiogram and echocardiography in the total patient population by country

Characteristic, *n* (%) or mean ± SD or median (IQR)	France (883 pts, 9 sites)	Spain (454 pts, 5 sites)	Germany (362 pts, 6 sites)	Italy (287 pts, 4 sites)	Austria (176 pts, 2 sites)	*p* value
Electrocardiogram						
Rhythm						< 0.001
Sinus rhythm	657 (77.8)	325 (71.7)	248 (70.5)	216 (78.5)	101 (58.4)	
Atrial fibrillation	125 (14.8)	97 (21.4)	74 (21.0)	44 (16.0)	50 (28.9)	
Pacemaker rhythm	53 (6.3)	31 (6.8)	27 (7.7)	12 (4.4)	14 (8.1)	
Other	10 (1.2)	0 (0.0)	3 (0.9)	3 (1.1)	8 (4.6)	
AV block second or third degree	4 (0.5)	6 (1.3)	3 (0.9)	2 (0.7)	1 (0.6)	0.544
Left bundle branch block	85 (10.0)	53 (11.7)	42 (11.9)	27 (9.8)	31 (17.9)	0.046
Right bundle branch block	116 (13.7)	48 (10.6)	37 (10.5)	34 (12.4)	14 (8.1)	0.168
Echocardiography						
LVEF < 50%	106 (12.5)	53 (11.7)	84 (24.6)	42 (14.8)	51 (29.0)	< 0.001
AR, mod/severe	122 (14.9)	54 (11.9)	56 (16.4)	43 (15.2)	12 (6.8)	0.020
AV mean PG (mmHg)	47.2 ± 12.8 45.0 (41.0, 53.0)	45.3 ± 11.8 44.5 (40.0, 51.0)	43.1 ± 16.4 41.0 (32.3, 53.0)	46.0 ± 13.6 44.0 (39.0, 53.0)	42.6 ± 14.0 41.5 (34.0, 48.8)	< 0.001
AV peak PG (mmHg)	71.6 ± 18.3 70.0 (61.0, 81.0)	74.6 ± 18.3 73.5 (65.0, 83.0)	69.5 ± 24.1 66.5 (54.0, 85.0)	72.8 ± 19.2 71.0 (62.5, 82.0)	71.3 ± 20.5 70.0 (60.0, 80.0)	< 0.001
*V*_max_ (m/s)	4.1 ± 0.9 4.2 (3.9, 4.5)	4.3 ± 0.6 4.4 (4.1, 4.7)	4.0 ± 0.8 4.0 (3.6, 4.4)	4.2 ± 0.8 4.2 (4.0, 4.5)	4.2 ± 0.6 4.2 (3.9, 4.4)	< 0.001
AVA (cm^2^)	0.8 ± 0.4 0.8 (0.6, 0.9)	0.8 ± 0.2 0.7 (0.6, 0.9)	0.7 ± 0.2 0.7 (0.6, 0.9)	0.7 ± 0.2 0.8 (0.6, 0.9)	0.8 ± 0.2 0.7 (0.6, 0.9)	0.002
Indexed AVA (cm^2^/m^2^)	0.4 ± 0.2 0.4 (0.4, 0.5)	0.4 ± 0.1 0.4 (0.4, 0.5)	0.4 ± 0.1 0.4 (0.3, 0.5)	0.4 ± 0.1 0.4 (0.3, 0.5)	0.4 ± 0.1 0.4 (0.3, 0.5)	< 0.001

*AR* aortic regurgitation, *AV* aortic valve, *AVA* aortic valve area, *IQR* interquartile range, *LVEF* left ventricular ejection fraction, *PG* pressure gradient, *SD* standard deviation, *pts* patients

**Fig. 1:**
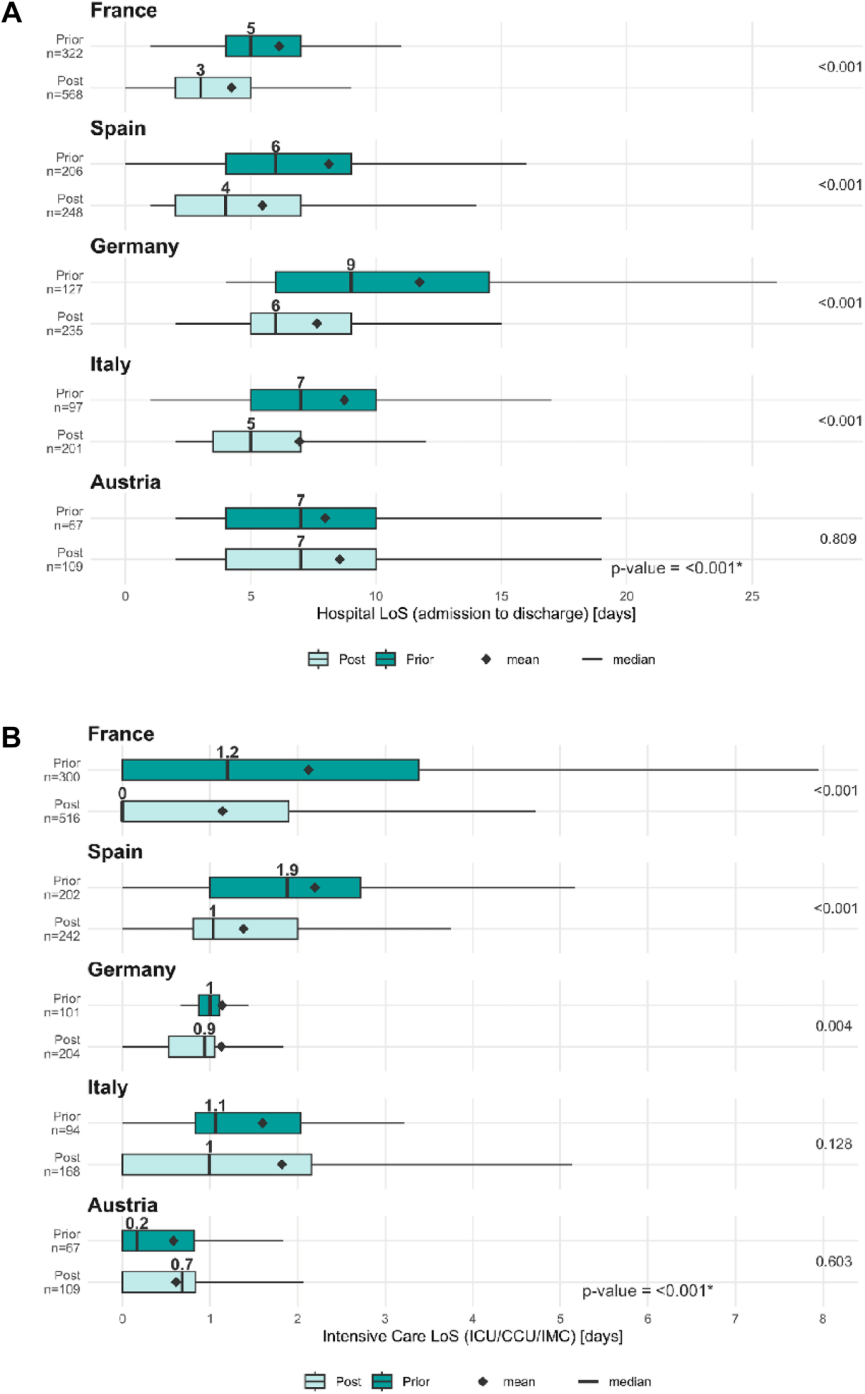
Hospital LoS (**A**) and intensive care LoS (**B**) prior to and post-Benchmark best practices implementation. Legend: *CCU* critical care unit, *ICU* intensive care unit, *IMC* intermediate care, *LoS* length of stay

After the implementation of Benchmark, French patients received local anaesthesia (LA) with minimal sedation or anxiolytic more often and had the shortest intervention time (median [interquartile range (IQR)] 55.0 [45.0, 70.5]), but had the highest rates of perioperative permanent pacemaker implantations (8.4%) compared to other countries (Table 3). There was a significant reduction in the hospital LoS in France after the implementation of Benchmark (from median 5.0 to 3.0 days, 40.0% decrease; *p* < 0.001) (Fig. 1A). At baseline, LoS in critical care was one of the highest in France (median 1.1 days) compared to other countries. After Benchmark implementation, France reported the highest reduction in the ICU/CCU/IMC LoS (from median 1.2 to 0 days (mean 2.1 to 1.1 days); *p* < 0.001) (Fig. 1B). Overall, the rates of complications at 30 days were low in France and remained unchanged before or after implementation of Benchmark (Table 4).

**Table 3:** Procedural details and outcomes post-Benchmark implementation by country

Characteristic, *n* (%) or mean ± SD or median (IQR)	France (568 pts, 9 sites)	Spain (248 pts, 5 sites)	Germany (235 pts, 6 sites)	Italy (202 pts, 4 sites)	Austria (109 pts, 2 sites)	*p* value
Local anaesthesia (LA) ± conscious sedation	544 (96.8)	224 (90.3)	231 (98.3)	177 (96.7)	104 (95.4)	< 0.001
LA w/o sedation/anxiolytic	76 (13.6)	2 (0.8)	41 (17.4)	18 (9.9)	0 (0.0)	
LA + minimal sedation/anxiolytic	292 (52.1)	98 (39.7)	94 (40.0)	87 (47.8)	1 (0.9)	
LA + moderate sedation	173 (30.9)	25 (10.1)	84 (35.7)	67 (36.8)	103 (94.5)	
Deep sedation	1 (0.2)	98 (39.7)	12 (5.1)	4 (2.2)	0 (0.0)	
Procedural information						
Total procedure time	46.2 ± 23.2 40.0 (30.0, 55.0)	73.0 ± 31.1 70.0 (50.0, 85.0)	46.4 ± 22.1 40.0 (32.0, 55.0)	87.4 ± 38.1 77.5 (60.0, 100.0)	41.5 ± 23.7 37.0 (24.0, 48.8)	< 0.001
Intervention time	63.9 ± 31.5 55.0 (45.0, 70.5)	106.3 ± 40.6 100.0 (80.0, 125.0)	88.7 ± 30.0 90.0 (65.0, 106.0)	111.6 ± 36.3 105.0 (90.0, 130.0)	80.4 ± 35.7 80.0 (46.0, 110.0)	< 0.001
Procedural success	546 (98.6)	247 (100.0)	229 (97.4)	180 (100.0)	109 (100.0)	0.017
Complications						
Second valve needed	4 (0.7)	0 (0.0)	2 (0.9)	0 (0.0)	0 (0.0)	0.528
Permanent pacemaker implantation	47 (8.4)	14 (5.6)	7 (3.0)	7 (3.8)	1 (0.9)	0.002
Conversion to conventional surgery	3 (0.5)	0 (0.0)	2 (0.9)	0 (0.0)	0 (0.0)	0.657
Bleeding^a^	9 (1.6)	15 (6.1)	1 (0.4)	3 (1.8)	1 (0.9)	0.002

*IQR* interquartile range, *LA* local anaesthesia, *SD* standard deviation, *pts* patients

^a^Major bleeding (BARC type 3a) defined as overt bleeding either associated with a drop in the haemoglobin level of at least 3.0 g/dL or requiring transfusion of 2 or 3 units of whole blood/RBC, or causing hospitalization or permanent injury, or requiring surgery AND does not meet criteria of life-threatening or disabling bleeding

**Table 4:** 30-day outcomes prior and post-Benchmark implementation by country

Outcome, *n* (%)		France (prior *n* = 322, post *n* = 568)	*p *value	Spain (prior *n* = 206, post *n* = 248)	*p *value	Germany (prior *n* = 127, post *n* = 235)	*p *value	Italy (prior *n* = 98, post *n* = 202)	*p *value	Austria (prior n = 67, post *n* = 109)	*p *value	*p *value^a^
Combined end point^b^	Prior	3 (1.1)	0.560	5 (2.4)	0.326	2 (1.7)	1.000	2 (2.2)	0.683	2 (3.4)	0.126	
	Post	10 (1.9)		10 (4.1)		5 (2.4)		4 (4.3)		0 (0.0)		0.094
All-cause mortality	Prior	0 (0.0)	0.305	1 (0.5)	1.000	0 (0.0)	0.531	1 (1.1)	0.492	0 (0.0)	–	
	Post	4 (0.8)		1 (0.4)		2 (1.0)		0 (0.0)		0 (0.0)		0.913
Stroke/TIA	Prior	2 (0.8)	1.000	3 (1.5)	0.518	2 (1.7)	1.000	2 (2.2)	0.683	1 (1.7)	0.358	
	Post	5 (1.0)		6 (2.5)		3 (1.4)		4 (4.2)		0 (0.0)		0.074
Life-threatening bleeding	Prior	2 (0.8)	0.606	5 (2.4)	0.439	1 (0.9)	1.000	2 (2.2)	1.000	1 (1.7)	1.000	
	Post	2 (0.4)		9 (3.7)		1 (0.5)		3 (3.2)		1 (0.9)		**0.002**
AKI (stage 2/3, incl. dialysis)	Prior	0 (0.0)	–	4 (2.0)	1.000	0 (0.0)	–	0 (0.0)	0.497	0 (0.0)	0.538	
	Post	0 (0.0)		4 (1.7)		0 (0.0)		2 (2.2)		2 (1.9)		**0.002**
Coronary obstruction requiring intervention	Prior	1 (0.4)	0.337	1 (0.5)	1.000	3 (2.6)	0.671	0 (0.0)	1.000	0 (0.0)	–	
	Post	0 (0.0)		1 (0.4)		3 (1.5)		1 (1.1)		0 (0.0)		0.033
Major vascular complications	Prior	6 (2.3)	0.603	9 (4.4)	0.937	7 (5.9)	** < 0.001**	6 (6.7)	0.528	4 (6.8)	0.056	
	Post	9 (1.7)		11 (4.5)		0 (0.0)		4 (4.3)		1 (0.9)		**0.004**
Re-hospitalization (all-cause)	Prior	8 (3.0)	0.746	9 (3.7)	0.650	2 (1.7)	0.716	0 (0.0)	1.000	2 (3.4)	0.618	
	Post	18 (3.5)		6 (2.9)		6 (2.9)		0 (0.0)		2 (1.9)		0.393

**Bold** formatting indicates statistical significance

*AKI* acute kidney injury, *TIA* transitory ischaemic attack, *pts* patients

^a^Cross-country comparison post-Benchmark implementation (Chi-squared or Fisher’s exact test)

^b^Includes all-cause mortality, stroke/TIA, and valve-related re-hospitalization[27]

### Spain

The highest body mass index (BMI, 28.0 ± 5.3 kg/m^2^) and rate of diabetes mellitus (42.7%) were reported in Spain (Table 1). Nevertheless, the Spanish cohort had the lowest reported surgical risk (EuroSCORE II 3.8 ± 2.6%). Echocardiographic data reported that patients treated in Spain had the highest mean aorto-ventricular peak PG (74.6 ± 18.3 mmHg) and AV maximal velocity (*V*_max_, 4.3 ± 0.6 m/s) (Table 2).

Peri-procedural deep sedation was routinely used for many patients in Spain (39.7%), in contrast to other countries, where more than 90% of patients received local anaesthesia with or without conscious sedation; vascular injury rates were also comparatively higher in Spain (6.1%) (Table 3). Spain also documented one of the longest procedure (median [IQR] 70.0 [50.0, 85.0 min]) and intervention (median [IQR] 100.0 [80.0, 125.0] min) times. The hospital LoS was significantly reduced in Spain following Benchmark (from median 6 to 4 days, 33.0% decrease; *p* < 0.001) (Fig. 1A). Similar to France, Spain initially reported the longest stay in critical care, which significantly decreased after the introduction of Benchmark (from median 1.9 to 1 day, 47.4% decrease; *p* < 0.001) (Fig. 1B). At 30 days, the rates of clinical events did not significantly change after the implementation of Benchmark (Table 4).

### Germany

Patients in Germany were characterized by a highly symptomatic presentation (NYHA class III/IV 64.9%), impaired mobility (27.6%), and the highest surgical risk (EuroSCORE II 6.8 ± 7.3%) at baseline (Table 1). The incidence of reduced left ventricular ejection fraction (LVEF < 50%) was high in the German cohort (24.6%) compared to France (12.5%), Spain (11.7%), and Italy (14.8%) (Table 2). Patients received LA without sedation or anxiolytic more often (17.7%) and had the lowest rate of intraprocedural bleeding complications (0.4%) (Table 3). Germany reported the longest hospital LoS prior to Benchmark and achieved the largest decrease after the implementation of Benchmark (from median 9.0 to 6.0 days, 33.0% decrease; *p* < 0.001), while critical care LoS was also significantly reduced (from median 1 to 0.9 days, 10.0% decrease; *p* = 0.004) (Fig. 1A and B). Furthermore, the implementation of Benchmark resulted in lower rates of major vascular complications at 30 days (5.9 vs 0.0%, *p* < 0.001) (Table 4).

### Italy

Italian patients had a high surgical risk (EuroSCORE II 6.0 ± 6.5%) and presented with higher rates of cardiac symptoms (NYHA class III/IV: 21.5%) and peripheral arterial disease (20.3%) compared to other cohorts. In contrast, their rate of impaired mobility was lowest (5.0%) (Table 1). Italian patients had the longest procedure (median [IQR] 77.5 [60.0, 100.0] min) and intervention times (median [IQR] 105 [90.0, 130.0] min) (Table 3). In Italy, there was a significant reduction in the median hospital LoS after the implementation of Benchmark (7 to 5 days, 29.0% decrease; *p* < 0.001), although the LoS in ICU/CCU/IMC did not change significantly (median 1.1 vs. 1 day, 9.0% decrease; *p* = 0.128) (Fig. 1A and B). The rates of 30-day events were unchanged following the implementation of Benchmark (Table 4).

### Austria

Patients in Austria reported the highest rates of dizziness with exertion (65.6%), (pre-)syncope (15.9%), and CCS angina class 3/4 (11.9%), and had the highest burden of prior myocardial infarction (29.0%, *p* < 0.001) and pulmonary hypertension (15.4%) (Table 1). Furthermore, patients in Austria had the highest rates of atrial fibrillation (28.9%), left bundle branch block (LBBB 17.9%) and LVEF < 50% (29.0%); overall, patients treated in Austria had lower surgical risk profiles than their European counterparts (EuroSCORE II 4.0 ± 3.8) (Table 2). Almost all patients (99.0%) received LA with moderate sedation; total procedure time was comparatively short (median [IQR] 37.0 [24.0, 48.8] minutes) (Table 3). Unlike in other countries, the implementation of Benchmark did not result in the reduction of either hospital LoS (median 7 vs 7 days, *p* = 0.809) or critical care LoS (0.2–0.7 days, *p* = 0.603) (Fig. 1A and B). The rate of major vascular complications was lower after the implementation of Benchmark (0.9% vs. 6.8%), although the difference did not reach statistical significance (*p* = 0.056) (Table 4).

## Discussion

The current analysis demonstrates that the implementation of Benchmark best practices resulted in reduced LoS while maintaining patient safety in different European countries despite differences in healthcare systems and patients’ baseline characteristics. The adoption of an evidence-based and expert-informed streamlined hospital pathway for TAVI patients supported with a peer mentorship programme can increase access to care, hospital capacity and efficiency in treating patients with severe AS in most European countries while optimizing outcomes.

Prior to the implementation of Benchmark, Germany reported the longest hospital LoS compared to other countries. The close scrutiny of processes of care and the implementation of bundle of best practices was instrumental in achieving a 33% decrease, the highest improvement among all reported countries. The mean hospital LoS in Germany prior to Benchmark was 11.7 days, which is comparable to the average TAVI hospital LoS reported in Germany between 2020 (11.5 days) and 2022 (11.1 days) [13]. Similarly, in Italy, the median hospital LoS prior to Benchmark in our report was 7 days, which corresponds to the results of the multicentre Italian OBSERVANT TAVI registry (median 7 days) [14]. After the implementation of Benchmark in the participating Italian centres, the median hospital LoS was significantly reduced by 2 days, resulting in a median of 5 days. As for the intensive care LoS, there was no reduction documented in Italy, which may be explained by the fact that the intensive care stay prior to Benchmark was already low compared to the intensive care duration reported in the OBSERVANT registry (median 1 vs. 2 days). In France, on the other hand, the highest reduction in the intensive care LoS was observed – from median 1.1 days prior to Benchmark to close to 0 days after Benchmark, meaning that the majority of patients did not stay on an intensified care unit at all after TAVI. The total hospital LoS was also reduced from median 5 to 3 days. The recent analysis of the FRANCE 2 and FRANCE-TAVI registries as well as the single-payer national health data system (SNDS) reported a median LoS of 5 days in the period between 2010 and 2021, which corresponds to the number observed in our registry prior to the implementation of Benchmark best practices [15]. In Spain, a significant decrease in the total hospital LoS (median 6 to 4 days) and intensive care LoS (median 1.7 to 1 day) was observed as well. The median hospital LoS reported in the Spanish TAVI registry was 6 days, which, once again, corresponds with the duration of hospital admission recorded in our registry prior to the introduction of Benchmark best practices [16]. Thus, patients in this registry documented prior to the implementation of Benchmark had a hospital LoS similar to multiple other registries, which accurately reflects real world practice and indicates the effectiveness of Benchmark best practices in reducing hospital LoS in these patient populations.

The reduction in the total hospital LoS was observed in all considered countries, except for Austria, where neither the length of hospital stay nor intensive care stay were reduced. Notably, Austrian patients tended to have a sicker profile at baseline compared to other countries. These patients tended to be more symptomatic and having prior myocardial infarction, pulmonary hypertension, atrial fibrillation, LBBB, and LVEF < 50% more often. Interestingly, this finding is not reflected in the EuroSCORE II, which appeared to be on lower side of the spectrum (4.0 ± 3.8). However, the characteristics of the current Austrian population are different when comparing these patients to those included in the nationwide Austrian TAVI registry as well as the recent AUTHEARTVISIT study [17, 18]. The reason for this inconsistency may be the fact that the Austrian TAVI registry included patient data from 11 centres for interventional cardiology representing 100% of Austrian institutions offering TAVI by vascular access sites, whereas the AUTHEARTVISIT study included patient data from the Austrian Insurance funds from 2010 through 2020, making the profile of the Austrian patient population reported in the current analysis less representative.

Besides the decrease in hospital LoS, one major goal of the Benchmark implementation was to preserve safety after TAVI. In our report, the 30-day safety was uncompromised in all countries included, which reinforces the results published for the total BENCHMARK population [10]. Moreover, there was no increase in the rate of hospital readmission documented after the implementation of Benchmark best practices, despite earlier discharge. There was also a significant reduction in the rates of major vascular complications in Germany after the implementation of Benchmark best practices (5.9% vs. 0.0%). The rates of all-cause mortality were quite low across all countries (< 1.0%) before and also with the implementation of Benchmark best practices compared to the previously reported rates in other registries. For instance, the 30-day mortality in the combined analysis of French TAVI registries was 4.0% compared to 0.0% and 0.8% prior to and after Benchmark implementation, respectively [15]. Similarly, the Italian OBSERVANT registry reported the rates of 30-day mortality to be 6.7% in males and 5.1% in females as opposed to 1.1% prior to and 0.0% after Benchmark in our registry [14]. Same differences are observed when comparing the 30-day mortality rates from the Spanish, German, and Austrian TAVI registries with the rates presented here [16, 18, 19]. Lower mortality rates in our patient population may be partially attributed to the lower risk profile of TAVI patients, better device technology with the use of new generation valves as well as increased operator experience.

Streamlining care by shortening hospital LoS offers a number of benefits, including minimizing hospital costs and resources related to unnecessary hospitalization time and the positive outcomes of early mobilization. Furthermore, the risk of in hospital-acquired infections and hospital readmission at 30 days is also reduced when patients are discharged earlier after TAVI [20, 21]. Based on our findings and other reports, the main drivers of accelerated recovery and shortened hospital LoS include various preventive measures, such as determination of an anticipated discharge based on pre-procedural risk stratification, the use of cardiac catheterization laboratory and local anaesthesia with or without light procedural sedation, removal of the temporary pacemaker at the end of the procedure, post-procedure echo- or angiographic check to confirm proper closing of access site, and the reduced use of critical care facilities [9, 22, 23]. In addition, patient and family engagement to facilitate shared decision-making plays a crucial role in early discharge. It is important to note, however, that several factors, such as cultural norms and the challenges of change management, should be considered for the wider adoption of Benchmark best practices across various countries. For example, local reimbursement issues related to the fixed minimum hospital stay duration, lack of resources, or certain attitudes and ideologies may result in stronger resistance to change. However, the use of contemporary evidence, peer-to-peer coaching and strong focus on quality improvement and access to care demonstrated in the BENCHMARK registry may serve as facilitators to the broader implementation of a streamlined patient pathway.

From the economical perspective, TAVI itself has become a superior approach in terms of lower costs and better outcomes compared with surgery and the shorted hospital LoS adds an additional economic benefit to that. In the propensity-matched economic analysis of the 3M-TAVR registry, the minimalist clinical pathway led to ≈$11,000 lower cumulative costs at 30 days, of which > $8000 were saved in non-procedure related costs during the index hospitalization, driven primarily by shorter LoS [24]. A shorter hospital stay also allows additional hospital revenue by freeing up hospital beds for other patients [24]. While LoS is the major contributor to TAVI costs, the avoidance of general anaesthesia, resulting in shorter procedure times, and minimal use of critical care services result in further savings [25, 26]. The planned economic analysis of the BENCHMARK registry will further assess potential costs savings associated with the implementation of best practices across different healthcare systems.

## Limitations

Although the present analysis successfully demonstrated the effectiveness of implementing Benchmark best practices across different European countries, it comes with limitations: This international, multicentre registry is purely observational, with the inherent limitations and potential bias on data collection, such as a higher potential for missing data. However, the observational design carries several advantages, including the assessment of TAVI patients in a real-world setting (high external validity), avoiding the existing issue of the strict inclusion and exclusion criteria used for randomized research (high internal, but less external validity). In addition, while the international nature of this study is highly advantageous and increases the applicability of our findings to other European countries, differences in the healthcare systems in each country (e.g., financial aspects, standard procedural and post-TAVI protocols, etc.) need to be considered when interpreting the findings. This was the reason for the present analysis. Lastly, the clinical outcomes were not independently adjudicated, which may have introduced a certain level of bias.

## Conclusion

The findings from the present analysis highlight that the implementation of Benchmark best practices in different European healthcare systems is equally effective in streamlining TAVI patient pathways by reducing hospital LoS without compromising on patient safety. Thus, the benefits of Benchmark best practices seem to extend beyond national borders, country peculiarities and patient profiles, primarily improving patients’ TAVI pathway. However, the breakdown of TAVI-specific parameters by country before and after implementation of Benchmark best practices enables to broaden its scope to more countries and centres. As a results, country specific measures are currently being developed to enable a further optimization and refinement of the TAVI pathway.

## Supplementary Information

Below is the link to the electronic supplementary material.

Supplementary file1 (DOCX 173 KB)

## Data Availability

Available from the corresponding author upon reasonable request.
