# Supplementary material for: The implementation of a streamlined TAVI patient pathway across five European countries: BENCHMARK registry
Source: Clin Res Cardiol. 2025 Apr 22;115(7):1107–19. doi: 10.1007/s00392-025-02638-z (PMC13249708; doi:10.1007/s00392-025-02638-z)
Supplement: Supplementary file 1 — Supplementary file1 (DOCX 173 KB) [file 392_2025_2638_MOESM1_ESM.docx]

**The implementation of a streamlined TAVI patient pathway across five European countries: BENCHMARK registry**

*Francesco Saia, MD; Sandra Lauck, PhD, RN; Eric Durand, MD; Douglas F Muir, MB ChB; Mark Spence, MD; Mariuca Vasa-Nicotera, MD; David Wood, MD; Cristóbal A. Urbano-Carrillo , MD; Damien Bouchayer , MD; Vlad Anton Iliescu , MD; Christophe Saint Etienne , MD; Florence Leclercq , MD; Vincent Auffret , MD; Lluis Asmarats , MD; Carlo Di Mario , MD; Aurelie Veugeois , MD, Jiri Maly , MD; Andreas Schober , MD; Luis Nombela-Franco , MD; Nikos Werner , MD; Joan Antoni Gómez-Hospital , MD; Julia Mascherbauer , MD; Giuseppe Musumeci , MD; Nicolas Meneveau , MD; Thibaud Meurice, MD; Felix Mahfoud , MD; Federico De Marco , MD, PhD; Tim Seidler , MD; Florian Leuschner , MD; Patrick Joly , MD; Jean Philippe Collet† , MD; Ferdinand Vogt , MD; Emilio Di Lorenzo , MD; Elmar Kuhn , MD; Vicente Peral Disdier , MD; Gemma McCalmont , RN; Radka Rakova , PhD; Wilbert Wesselink , PhD; Jana Kurucova ,* *MD; Violetta Hachaturyan , MSc; Claudia M. Lüske , PhD; Peter Bramlage, MD; Derk Frank , MD for the BENCHMARK Investigator Group*

**Corresponding author**

Prof. Dr. Derk Frank

Department of Internal Medicine III (Cardiology and Critical Care Medicine), University Clinical Centre Schleswig-Holstein (UKSH) &

German Centre for Cardiovascular Research, partner site Hamburg/Kiel/Lübeck

Arnold-Heller Strasse 3, 24105 Kiel, Germany

Phone: +49 431 500-22801

Email: derk.frank@uksh.de

**SUPPLEMENT**

**Supplementary Table 1:** Patient characteristics by country and by phase

|  |  | **France**  **(Prior n=322;**  **Post n=568)** | *p-value* | **Spain**  **(Prior n=206;**  **Post n=248)** | *p-value* | **Germany**  **(Prior n=127;**  **Post n=235)** | *p-value* | **Italy**  **(Prior n=98;**  **Post n=202)** | *p-value* | **Austria**  **(Prior n=67;**  **Post n=109)** | *p-value* |
| --- | --- | --- | --- | --- | --- | --- | --- | --- | --- | --- | --- |
| Age (years) | Prior | 79.6 ± 7.2 | 0.180 | 80.7 ± 6.2 | 0.949 | 79.9 ± 6.3 | 0.450 | 81.3 ± 5.0 | 0.362 | 79.9 ± 6.6 | 0.824 |
|  | Post | 80.4 ± 6.9 |  | 80.7 ± 6.6 |  | 79.4 ± 6.8 |  | 81.8 ± 4.8 |  | 80.2 ± 6.2 |  |
| Female gender | Prior | 116 (36.0) | 0.189 | 99 (48.1) | 0.011 | 54 (42.5) | 0.094 | 44 (45.4) | 0.909 | 28 (41.8) | 0.119 |
|  | Post | 228 (40.5) |  | 90 (36.3) |  | 79 (33.6) |  | 88 (46.1) |  | 33 (30.3) |  |
| BMI (kg/m^2^) | Prior | 27.8 ± 5.3 | 0.499 | 28.3 ± 5.1 | 0.098 | 27.3 ± 4.4 | 0.460 | 27.2 ± 4.9 | 0.354 | 28.0 ± 4.9 | 0.899 |
|  | Post | 27.5 ± 4.9 |  | 27.7 ± 5.4 |  | 27.0 ± 4.6 |  | 26.6 ± 4.4 |  | 27.8 ± 4.8 |  |
| Dizziness | Prior | 21 (6.6) | 0.888 | 106 (51.5) | 0.631 | 23 (18.1) | 0.715 | 28 (29.2) | 0.024 | 38 (56.7) | 0.135 |
|  | Post | 35 (6.3) |  | 122 (49.2) |  | 39 (16.6) |  | 32 (17.5) |  | 74 (67.9) |  |
| (Pre-)syncope | Prior | 16 (5.0) | 0.366 | 26 (12.6) | 0.567 | 10 (7.9) | 0.157 | 9 (9.4) | 0.504 | 9 (13.4) | 0.481 |
|  | Post | 36 (6.5) |  | 27 (10.9) |  | 30 (12.8) |  | 22 (12.0) |  | 19 (17.4) |  |
| NYHA class III or IV | Prior | 181 (56.7) | 0.008 | 122 (59.2) | 0.552 | 79 (63.7) | 0.731 | 60 (62.5) | 0.764 | 47 (70.1) | 0.046 |
|  | Post | 262 (47.4) |  | 140 (56.5) |  | 154 (65.5) |  | 111 (60.7) |  | 60 (55.0) |  |
| Angina CCS 3 or 4 | Prior | 11 (3.4) | 0.052 | 11 (5.3) | 0.103 | 7 (5.6) | 0.329 | 8 (8.3) | 0.908 | 11 (16.4) | 0.150 |
|  | Post | 8 (1.4) |  | 6 (2.4) |  | 8 (3.4) |  | 16 (8.7) |  | 10 (9.2) |  |
| EuroSCORE II | Prior | 5.2 ± 5.7 | <0.001 | 4.4 ± 2.9 | <0.001 | 6.8 ± 6.3 | 0.409 | 5.3 ± 4.7 | 0.469 | 3.7 ± 2.5 | 0.506 |
|  | Post | 4.2 ± 7.0 |  | 3.3 ± 2.3 |  | 6.8 ± 7.9 |  | 6.3 ± 7.2 |  | 4.2 ± 4.4 |  |
| Frailty, severe | Prior | 7 (2.2) | 0.110 | 20 (9.7) | 0.008 | 6 (4.7) | 0.742 | 2 (2.1) | 1.000 | 2 (3.0) | 0.636 |
|  | Post | 4 (0.7) |  | 9 (3.6) |  | 13 (5.5) |  | 3 (1.6) |  | 2 (1.8) |  |
| Impaired mobility | Prior | 25 (7.8) | 0.077 | 41 (19.9) | 0.100 | 27 (21.3) | 0.046 | 6 (6.3) | 0.565 | 19 (28.4) | 0.002 |
|  | Post | 27 (4.8) |  | 35 (14.1) |  | 73 (31.1) |  | 8 (4.3) |  | 11 (10.1) |  |
| Cognitive deficit | Prior | 6 (1.9) | 0.550 | 12 (5.8) | 0.501 | 6 (4.7) | 0.985 | 4 (4.2) | 0.740 | 4 (6.0) | 0.203 |
|  | Post | 14 (2.5) |  | 11 (4.4) |  | 11 (4.7) |  | 6 (3.3) |  | 2 (1.8) |  |
| Prior MI | Prior | 34 (10.6) | 0.946 | 37 (18.0) | 0.774 | 14 (11.0) | 0.801 | 15 (15.6) | 0.380 | 14 (20.9) | 0.064 |
|  | Post | 58 (10.4) |  | 42 (16.9) |  | 28 (11.9) |  | 22 (11.9) |  | 37 (33.9) |  |
| PAD | Prior | 80 (24.8) | <0.001 | 18 (8.7) | 0.627 | 11 (8.7) | 0.961 | 19 (19.8) | 0.882 | 11 (16.4) | 0.744 |
|  | Post | 85 (15.3) |  | 25 (10.1) |  | 20 (8.5) |  | 38 (20.5) |  | 20 (18.3) |  |
| Diabetes mellitus | Prior | 83 (25.8) | 0.887 | 97 (47.1) | 0.087 | 42 (33.1) | 0.465 | 23 (24.0) | 0.456 | 20 (29.9) | 0.127 |
|  | Post | 146 (26.2) |  | 97 (39.1) |  | 69 (29.4) |  | 52 (28.1) |  | 45 (41.3) |  |
| PH, severe* | Prior | 13 (4.8) | 0.133 | 12 (6.4) | 0.545 | 9 (7.1) | 0.399 | 6 (6.5) | 0.746 | 7 (13.5) | 0.615 |
|  | Post | 13 (2.7) |  | 18 (7.9) |  | 22 (9.7) |  | 11 (7.6) |  | 14 (16.7) |  |
| Renal insufficiency | Prior | 80 (24.8) | 0.720 | 61 (29.6) | 0.006 | 32 (25.2) | 0.739 | 30 (31.3) | 0.940 | 18 (26.9) | 0.628 |
|  | Post | 145 (25.9) |  | 46 (18.5) |  | 63 (26.8) |  | 57 (30.8) |  | 33 (30.3) |  |
| Prior pacemaker | Prior | 28 (9.0) | 0.638 | 10 (4.9) | 0.010 | 12 (12.5) | 0.944 | 6 (6.4) | 0.910 | 4 (8.2) | 0.731 |
|  | Post | 43 (8.1) |  | 29 (11.8) |  | 28 (12.8) |  | 11 (6.7) |  | 6 (6.1) |  |

*Legend:* BMI, body mass index; CCS, Canadian Cardiovascular Society; IQR, interquartile range; MI, myocardial infarction; NYHA, New York Heart Association; SD, standard deviation; PAD, peripheral arterial disease; pts, patients; PH pulmonary hypertension

* Mean pulmonary arterial pressure >55 mmHg

**Supplementary Table 2:** Length of stay on critical care (ICU/CCU/IMC) by country and by phase

|  |  | **France**  **(Prior n=300;**  **Post n=516)** | *p-value* | **Spain**  **(Prior n=202;**  **Post n=242)** | *p-value* | **Germany**  **(Prior n=101;**  **Post n=204)** | *p-value* | **Italy**  **(Prior n=94;**  **Post n=168)** | *p-value* | **Austria**  **(Prior n=67;**  **Post n=109)** | *p-value* |
| --- | --- | --- | --- | --- | --- | --- | --- | --- | --- | --- | --- |
| LoS in ICU/CCU/IMC, days | Prior | 2.1 ± 3.1  1.2 (0.0, 3.4) | <0.001 | 2.2 ± 2.1  1.9 (1.0, 2.7) | <0.001 | 1.1 ± 0.7  1.0 (0.9, 1.1) | 0.004 | 1.6 ± 1.6  1.1 (0.8, 2.0) | 0.128 | 0.6 ± 0.9  0.2 (0.0, 0.8) | 0.603 |
|  | Post | 1.1 ± 1.8  0.0 (0.0, 1.9) |  | 1.4 ± 1.3  1.0 (0.8, 2.0) |  | 1.1 ± 1.6  0.9 (0.5, 1.1) |  | 1.8 ± 2.8  1.0 (0.0, 2.2) |  | 0.6 ± 0.9  0.7 (0.0, 0.8) |  |

*Legend: LoS, length of stay*

**Supplementary Table 3:** Total length of stay by country and by phase

|  |  | **France**  **(Prior n=322;**  **Post n=568)** | *p-value* | **Spain**  **(Prior n=206;**  **Post n=248)** | *p-value* | **Germany**  **(Prior n=127;**  **Post n=235)** | *p-value* | **Italy**  **(Prior n=97;**  **Post n=202)** | *p-value* | **Austria**  **(Prior n=67;**  **Post n=109)** | *p-value* |
| --- | --- | --- | --- | --- | --- | --- | --- | --- | --- | --- | --- |
| Total LoS, days | Prior | 6.1 ± 6.9  5.0 (4.0, 7.0) | <0.001 | 8.1 ± 7.4  6.0 (4.0, 9.0) | <0.001 | 11.7 ± 8.1  9.0 (6.0, 14.5) | <0.001 | 8.7 ± 6.1  7.0 (5.0, 10.0) | <0.001 | 8.0 ± 4.4  7.0 (4.0, 10.0) | 0.809 |
|  | Post | 4.2 ± 2.8  3.0 (2.0, 5.0) |  | 5.5 ± 5.7  4.0 (2.0, 7.0) |  | 7.6 ± 6.3  6.0 (5.0, 9.0) |  | 6.9 ± 6.6  5.0 (3.5, 7.0) |  | 8.6 ± 8.5  7.0 (4.0, 10.0) |  |

*Legend: LoS, length of stay*

**Supplementary Figure 1:** Benchmark best practices implementation rates (%) by country

|  |
| --- |
| **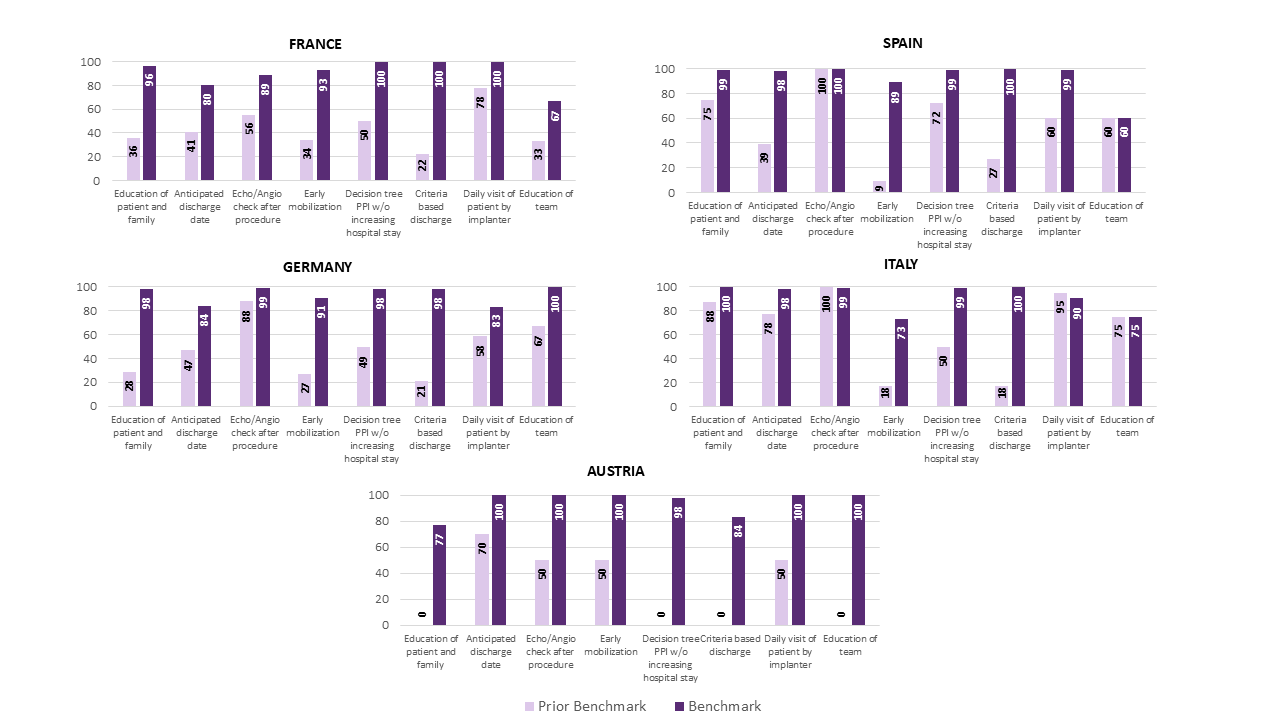** |
